# miR-143 Inhibits NSCLC Cell Growth and Metastasis by Targeting Limk1

**DOI:** 10.3390/ijms150711973

**Published:** 2014-07-07

**Authors:** Hui Xia, Shengjie Sun, Bo Wang, Tao Wang, Chaoyang Liang, Guo Li, Chongbiao Huang, Daliang Qi, Xiangyang Chu

**Affiliations:** 1 Department of Thoracic-Cardio Surgery, the First Affiliated Hospital of PLA General Hospital, Beijing 100048, China; E-Mail: hui_xiaa@126.com; 2 Department of Medical Oncology, the General Hospital of People’s Liberation Army, Beijing 100853, China; E-Mail: shengjie_sunn@126.com; 3 Department of Thoracic Surgery, the General Hospital of People’s Liberation Army, Beijing 100853, China; E-Mails: wang_boa@126.com (B.W.); wang_taow@126.com (T.W.); liang_cyc@126.com (C.L.); li_gguo@126.com (G.L.); 4 Key Laboratory of Cancer Prevention and Therapy of Tianjin, Department of Senior Ward, Tianjin Medical University Cancer Institute and Hospital, Huanhu Xilu, Hexi District, Tianjin 300060, China; E-Mail: huang_chongbiao@126.com

**Keywords:** miR-143, NSCLC, Limk1, proliferation, migration, invasion

## Abstract

MicroRNAs (miRNAs) have essential roles in carcinogenesis and tumor progression. Here, we investigated the roles and mechanisms of miR-143 in non-small cell lung cancer (NSCLC). miR-143 was significantly decreased in NSCLC tissues and cell lines. Overexpression of miR-143 suppressed NSCLC cell proliferation, induced apoptosis, and inhibited migration and invasion *in vitro*. Integrated analysis identified LIM domain kinase 1 (Limk1) as a direct and functional target of miR-143. Overexpression of Limk1 attenuated the tumor suppressive effects of miR-143 in NSCLC cells. Moreover, miR-143 was inversely correlated with Limk1 expression in NSCLC tissues. Together, our results highlight the significance of miR-143 and Limk1 in the development and progression of NSCLC.

## 1. Introduction

Non-small cell lung cancer (NSCLC), including adenocarcinoma and squamous cell carcinoma, is the leading cause of cancer deaths worldwide [1]. Despite an increasing number of studies on NSCLC genomics as well as the development of targeted therapies, the overall 5-year survival rate is only 15%. This highlights the need for a better understanding of NSCLC biology to improve the prevention, diagnosis, and treatment of this cancer [2]. Recently, evidence has shown that microRNAs (miRNAs) may be involved in NSCLC pathogenesis, providing novel insights into disease biology.

miRNAs are a class of small noncoding RNAs approximately 22 nucleotides in length that suppress gene expression by post-transcriptional mechanisms. MiRNAs bind to a complementary sequence in the 3'-untranslated regions (3'-UTR) of target mRNAs, resulting in either mRNA decay or inhibition of translation [3,4]. Emerging evidence has shown that miRNAs are associated with a variety of human cancers and act as both oncogenes and tumor suppressors [5,6]. In NSCLC, multiple tumor-suppressive miRNAs have been identified, such as miR-143, miR-197, and miR-503 [7,8,9]. Further miRNAs have been found to promote carcinogenesis and include miR-92b, miR-96, and miR-150 [10,11,12]. Therefore, the aberrant expression of miRNAs could provide a novel diagnostic strategy for cancer.

miR-143 belongs to the miR-143/145 cluster, which acts as a suppressor in several tumors [13,14]. Recently, miRNA profiling studies have indicated that miR-143 is decreased in several tumor tissues, including NSCLC [15,16,17]. Furthermore, increasing evidence suggests that miR-143 plays important roles in tumor pathogenesis via the targeting of different genes [18,19]. In this study, we demonstrate that decreased miR-143 expression is a characteristic molecular signature in NSCLC and that miR-143 may function as a tumor suppressor by directly targeting LIM domain kinase 1 (Limk1).

## 2. Results

### 2.1. miR-143 Is Downregulated in Non-Small Cell Lung Cancer (NSCLC) Tissues and Cell Lines

**Figure 1:**
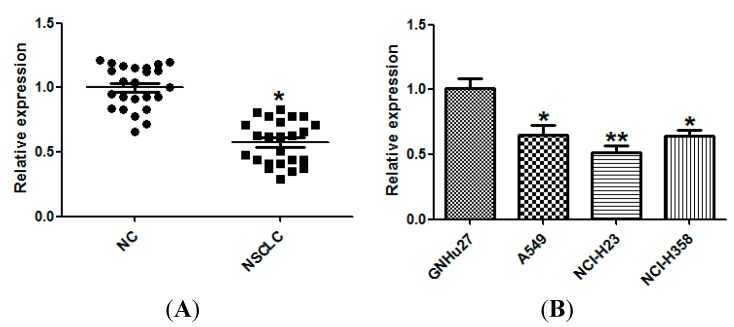
Down-regulation of miR-143 in non-small cell lung cancer (NSCLC) tissues and cell lines. (**A**) The expression of miR-143 in 24 pairs of NSCLC tissues and the matched normal lung tissues (NC) was measured by quantitative real time PCR (qRT-PCR); and (**B**) The expression of miR-143 in normal lung epithelial cell line (GNHu27) and 3 NSCLC cell lines (A549, NCI-H23, and NCI-H358) was measured by qRT-PCR. * *p* < 0.05, ** *p* < 0.01 compared with control.

To study the expression of miR-143 in NSCLC, we measured the expression of miR-143 in 24 pairs of NSCLC and matched normal lung tissues using quantitative real time PCR (qRT-PCR). The results showed that miR-143 expression was significantly down-regulated in NSCLC tissues compared with matched controls (Figure 1A). In addition, the expression of miR-143 in three NSCLC cell lines was determined, and was found to be substantially decreased compared with that in the normal cell line GNHu27 (Figure 1B). The clinicopathological features of 24 pairs of NSCLC patients are shown in Table 1, and miR-143 was correlated with the metastasis of NSCLC patients.

**Table 1:** Relationship between miR-143 and clinicopathological variables in NSCLC tissues.

Variable	*N*	Low	High	*p* Value
**Age (years)**				0.161 ^a^
≥60	14	8	6	
<60	10	6	4	
**Gender**				0.542 ^a^
Male	11	6	5	
Female	13	7	6	
**Size**				0.231 ^a^
>3 cm	14	8	6	
≤3 cm	10	4	6	
**Histology type**				0.416 ^a^
Adenocarcinoma	13	6	7	
Squamous cancer	11	6	5	
**Histological grade**				0.214 ^b^
I	11	5	7	
II	6	4	2	
III	7	4	3	
**Lymph node status**				0.024 ^a^
Metastasis	13	9	4	
No Metastasis	11	3	8	

^a^ Chi-square Test; ^b^ Mann-whitney Test.

### 2.2. miR-143 Suppresses the Proliferation of NSCLC Cells

**Figure 2:**
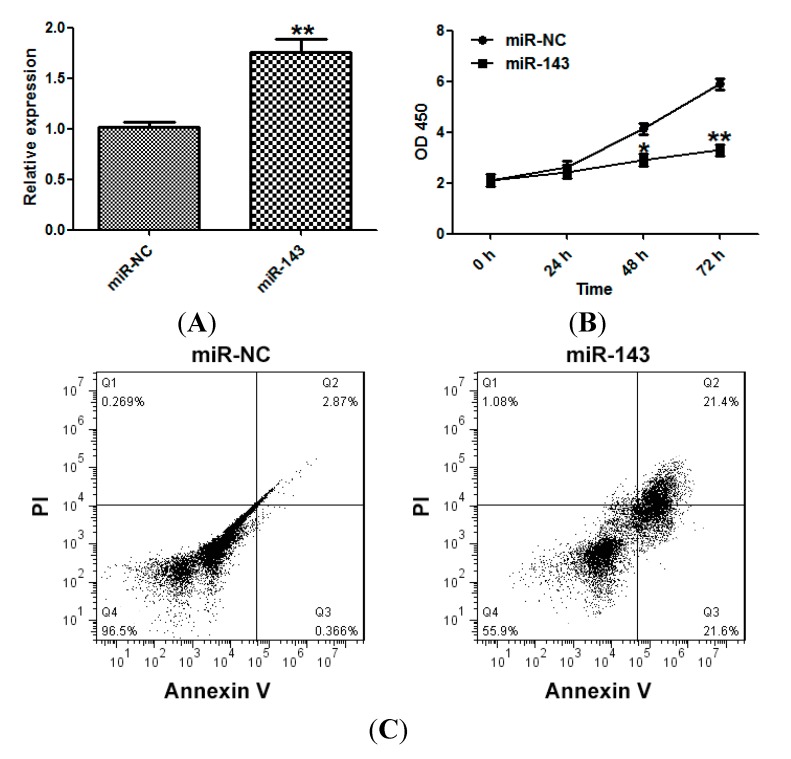
miR-143 suppresses the proliferation of NSCLC cells. (**A**) NCI-H23 cells were transfected with miR-143 or control mimic (miR-NC), and the expression of miR-143 was measured by qRT-PCR; (**B**) Cell viability assay (CCK-8); and (**C**) Apoptosis assays. Experiments were performed in triplicate. * *p* < 0.05, ** *p* < 0.01 compared with control.

To better understand the role of miR-143 in the development of NSCLC, we transfected NCI-H23 cells with miR-143 or a mimic control. Overexpression of miR-143 was confirmed by qRT-PCR (Figure 2A). The effect of miR-143 on NSCLC cell proliferation was examined using the Cell Counting Kit-8 (CCK-8) assay, and miR-143 was found to suppress the proliferation of NCI-H23 cells (Figure 2B). miR-143 overexpression also induced apoptosis in NCI-H23 cells (Figure 2C).

### 2.3. miR-143 Suppresses Migration and Invasion of NSCLC Cells

**Figure 3:**
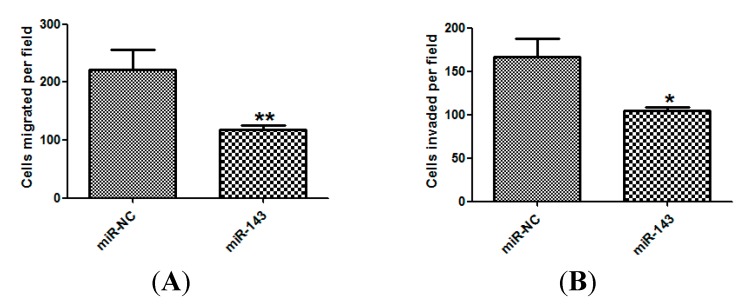
miR-143 suppresses migration and invasion of NSCLC cells. The migration (**A**) and invasion assays (**B**) of NCI-H23 cells transfected with miR-143 or miR-NC. Experiments were performed in triplicate. * *p* < 0.05, ** *p* < 0.01 compared with control.

We further investigated whether miR-143 could inhibit migration and invasion of NSCLC. Using the Transwell migration assay, we found that overexpression of miR-143 dramatically suppressed mobility in NCI-H23 tumor cells compared with the corresponding controls (Figure 3A,B).

### 2.4. LIM Domain Kinase 1 (Limk1) Is a Direct Target of miR-143 in NSCLC Cells

**Figure 4:**
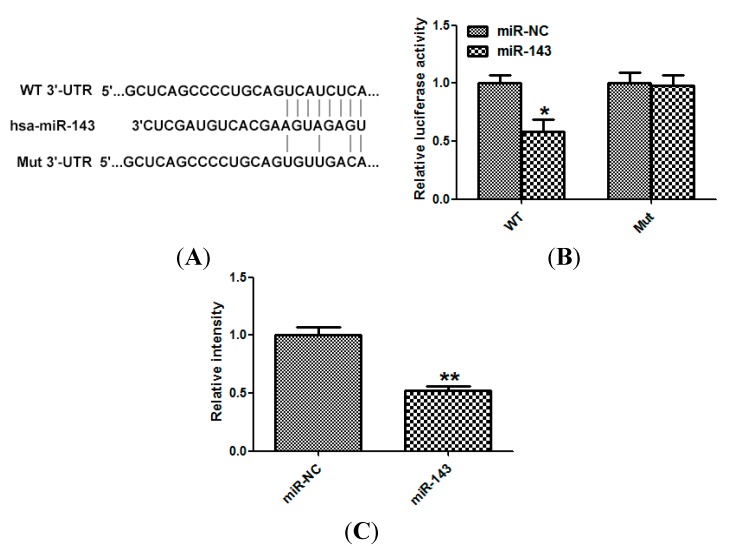
LIM domain kinase 1 (Limk1) is a direct target of miR-143 in NSCLC cells. (**A**) wild type Limk1 3'-untranslated regions (3'-UTR) (WT) or Mutated Limk1 3'-UTR (Mut) of Limk1 sequence; (**B**) HEK-293 cells were co-transfected with miR-143/miR-NC with WT/Mut 3'-UTR of Limk1. Relative luciferase activity was assayed; and (**C**) NCI-H23 cells transfected with miR-143 or miR-NC, and Western blot was used to detect the protein level of Limk1. Glyceraldehyde-3-phosphate dehydrogenase (GAPDH) was used as control. Experiments were performed in triplicate. * *p* < 0.05, ** *p* < 0.01 compared with control.

To investigate the downstream target of miR-143, TargetScan 6.2 was used [9]. Limk1 was predicted to be a target of miR-143 (Figure 4A). miR-143 suppressed the luciferase activity of the wild type but not the mutant 3'-UTR in HEK-293 cells (Figure 4B). Moreover, treatment with the miR-143 mimic decreased the protein level of Limk1 (Figure 4C).

### 2.5. miR-143 Suppresses NSCLC Development by Targeting Limk1

**Figure 5:**
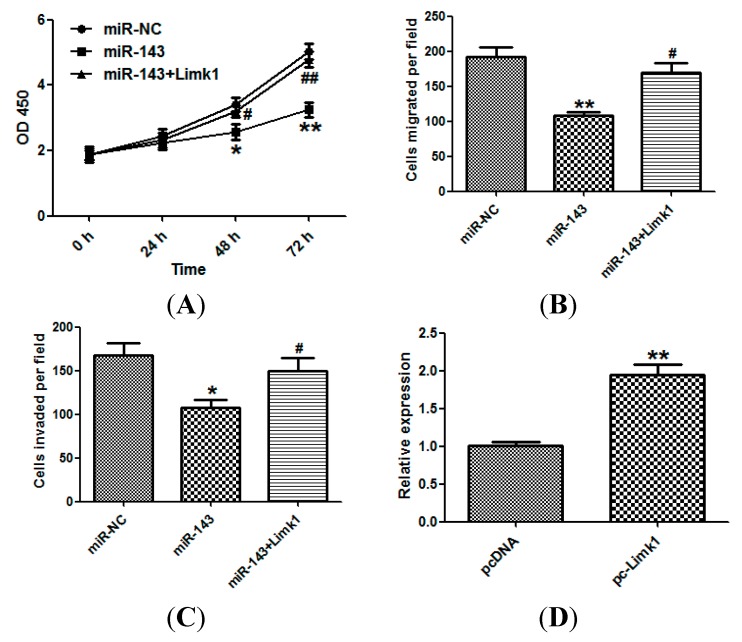
miR-143 suppresses NSCLC development by targeting Limk1. (**A**) NCI-H23 cells were transfected with miR-143 with/without Limk1 overexpression plasmid (pcDNA-Limk1), and CCK-8 assay was performed; (**B**) Migration and (**C**) invasion assays of NCI-H23 cells; and (**D**) The expression of Limk1 of cells transfected with pcDNA-Limk1 or control vector was detected by qRT-PCR. The data represented three independent experiments. Experiments were performed in triplicate. * *p* < 0.05, ** *p* < 0.01 compared with control; # *p* < 0.05, ## *p* < 0.01 compared with miR-143 transfected group.

The involvement of Limk1 in miR-143-induced suppression of NSCLC cell growth and invasion was examined using the CCK-8 assay (Figure 5A), migration (Figure 5B), and invasion (Figure 5C). In each case, Limk1 overexpression significantly attenuated the suppressive effects of miR-143 in NSCLC cells. The effect of Limk1 overexpression was confirmed by qRT-PCR (Figure 5D).

### 2.6. miR-143 Expression Is Inversely Correlated with Limk1 Expression in NSCLC Tissues

**Figure 6:**
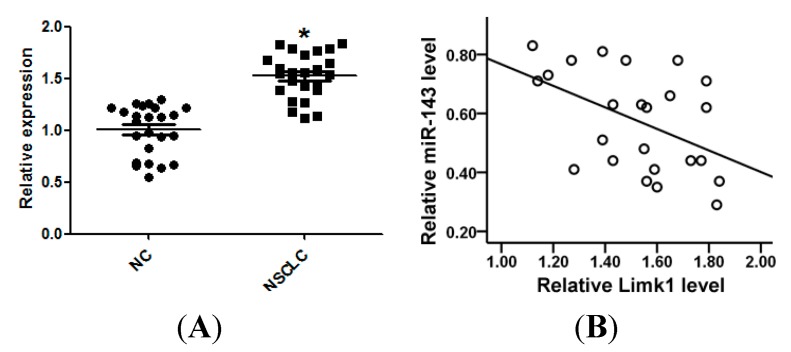
miR-143 is negatively correlated with Limk1 in NSCLC tissues. (**A**) The expression of Limk1 in 24 pairs of NSCLC tissues and the matched NC was measured by qRT-PCR; and (**B**) Limk1 mRNA level was inversely correlated with miR-143 level in NSCLC tissues (Spearman’s correlation analysis, *r* = −0.503, *p* = 0.012). * *p* < 0.05 compared with control.

Expression of Limk1 in 24 samples of NSCLC tissue, and the corresponding normal controls, was examined by qRT-PCR. Limk1 mRNA was markedly increased in NSCLC tissues (Figure 6A). Furthermore, the level of Limk1 mRNA expression was inversely correlated with that of miR-143 NSCLC tissues (Figure 6B).

## 3. Discussion

miRNAs have been reported to play critical roles in carcinogenesis and tumor progression [20,21]. Aberrant miRNA expression has been implicated in almost all aspects of tumor biology, including proliferation, apoptosis, migration, and invasion, and they can act as either tumor suppressors or oncogenes. Here, we focused on miR-143, which acts as a tumor suppressor in human malignancies. Ng *et al.* reported that miR-143 was significantly down-regulated in breast cancer, and that its overexpression suppressed proliferation and soft agar colony formation of breast cancer cells by down-regulating DNA methyltransferase 3A (DNMT3A) expression [15]. Zeng *et al*. reported that miR-143 down-regulation in peripheral blood mononuclear cells could be used as a novel diagnostic biomarker for NSCLC carcinogenesis [22]. Zhang *et al.* found that miR-143 regulated NSCLC cell apoptosis by inhibiting PKCε [18], and Ma *et al.* reported that miR-143 inhibited migration and invasion of NSCLC cells through the targeting of CD44v3 [19].

miR-143 and miR-145 are co-expressed miRNAs which form a bicistronic cluster in 5q33.1, and have been widely studied as potential tumor suppressors [23]. Viana *et al.* reported that miR-143 and miR-145 might be involved in the pathogenesis of benign prostatic hyperplasia via regulating target genes and proteins [24]. Kojima *et al.* found that in prostate cancer miR-143/145 cluster suppressed cell migration and invasion by targeting Golgi membrane protein 1 (GOLM1) [13]. Zhang *et al.* showed that down-regulation of miR-143 and miR-145 might be associated with overexpression of DNA methyltransferase 3B (DNMT3B) and worse prognosis in endometrioid carcinomas [25]. Cho *et al.* revealed that restoration of miR-145 suppressed cancer cell growth in lung adenocarcinoma patients who contained epidermal growth factor receptor (EGFR) mutation [26]. These studies suggest that miR-143 and miR-145 may co-contribute to the progression of several cancers. In NSCLC, miR-143 and miR-145 have been found to suppress the progression of NSCLC by targeting different targets [18,19,27]. Thus, whether miR-143 and miR-145 may co-contribute to the progression of NSCLC needs further studies.

In the present study, we showed that miR-143 was significantly down-regulated in NSCLC tissues and cell lines. Forced overexpression of miR-143 effectively suppressed NSCLC cell proliferation, enhanced apoptosis, and inhibited migration and invasion. Limk1 is a key regulator of the actin cytoskeleton, cell motility, and invasion, and is thought to be a therapeutic target for metastatic disease [28]. Limk1 is frequently overexpressed in many malignancies and functions as an important oncogene [9,29,30]. Downregulation of Limk1 suppressed migration of NSCLC cells and enhanced their sensitivity to chemotherapy drugs [29]. Increased Limk1 expression has been found in prostate tumor tissues, and is involved in regulating the invasiveness of prostate cancer cells [30]. Here, we demonstrate that Limk1 is a direct target of miR-143, which we confirm by luciferase activity and western blot. We found that the tumor suppressive effects of miR-143 on NSCLC cells were partially reversed by overexpression of Limk1. Finally, we have shown that Limk1 is significantly elevated in NSCLC tissues and its expression is inversely correlated with the level of miR-143 expression. Together, these data suggest that miR-143 inhibits NSCLC growth and metastasis in part through the down-regulation of Limk1.

In conclusion, the present study showed that miR-143 was significantly down-regulated in NSCLC tissues and cell lines. Forced overexpression of miR-143 inhibited tumor growth and metastasis of NSCLC cells partially through targeting Limk1.

## 4. Materials and Methods

### 4.1. Clinical Samples and Cell Lines

Paired NSCLC and corresponding noncancerous lung tissues were obtained from 24 consecutive patients with informed consent in our department. Tissues were snap-frozen in liquid nitrogen. NSCLC cell lines A549, NCI-H23 and NCI-H358 cells, and normal GNHu27 cells were purchased from ATCC. The cell lines were maintained in RPMI-1640 medium containing 10% FBS at 37 °C in a humidified atmosphere containing 5% CO_2_. Transfection was performed using Lipofectamine 2000 (Invitrogen, Carlsbad, CA, USA) according to the manufacturer’s protocol.

### 4.2. RNA Preparation and Quantitative Real Time PCR (qRT-PCR)

miRNAs were isolated using mir-Vana miRNA Isolation Kit from Ambion (Austin, TX, USA). Total RNAs were isolated using Trizol reagent (Invitrogen, Carlsbad, CA, USA). SYBR Green qRT-PCR was performed on ABI Stepone Plus (ABI, Foster City, CA, USA). The relative expression levels of genes were calculated using the 2^−ΔΔ*C*t^ method. Expression of Limk1 was normalized with Glyceraldehyde-3-phosphate dehydrogenase (GAPDH), and the level of miR-143 was normalized with U6. The primers for Limk1: Sense 5'-AGACCTCAACTCCCACAA-3' and antisense 5'-CTCAGGTGCCATCCAGT-3'.

### 4.3. Plasmid Construction

miR-143 and control mimics were obtained from RiboBio (Guangzhou, China). Limk1 cDNA was amplified and inserted into pcDNA3.1. The wild-type Limk1 3'-UTR was amplified with the following primers: Sense 5'-CCCTCGAGCCAGCAACCCTGTTCACG-3' and antisense 5'-TTGCGGCCGCCAACTGAGGCAAAGTGACAAA-3'. The PCR fragment was cloned into psiCHECK-2 vector within XhoI and NotI (Promega, Madison, WI, USA). Mutation of Limk1 3'-UTR was performed using a fast mutation kit (NEB, Ipswich, MB, Canada).

### 4.4. Cell Proliferation and Apoptosis Assays

The effect of miR-143 expression on NCI-H23 cell proliferation was assessed using CCK-8 kit (Dojindo, Kumamoto, Japan). Briefly, 5 × 10^3^ transfected cells were seeded in 96-well plates and cultured for 48 h. Exactly 10 μL WST-8 was added to each well and further incubated for 1 h at 37 °C. OD_450_ was measured using a microplate reader (Molecular Deviced, Sunnyvale, CA, USA).

Cells were washed with PBS and fixed with 70% ethanol. The percentage of apoptotic cells was measured by flow cytometry using the Annexin V/FITC and PI Apoptosis Detection Kit (Becton Dickinson, Cockeysville, MD, USA) according to the manufacturer instructions.

### 4.5. Cell Migration and Invasion Assays

For migration assay, 5 × 10^4^ cells resuspended in serum-free RPMI-1640 after transfection were seeded in the top portion of an 8-μm pore Transwell chamber (Millipore, Hayward, CA, USA). The lower chamber contained 10% FBS as a chemoattractant. Cells were incubated for 24 h. Non-migrating cells on the top surface of the membrane were removed, while cells which had migrated to the bottom surface of the membrane were fixed with 95% ethanol, stained with 0.1% crystal violet, and counted. Four random fields were analyzed for each chamber.

Matrigel-coated transwell were used for invasion assay. After 48 h, cells which had invaded to the lower surface of membrane were fixed, stained, and counted. Four random fields were analyzed for each chamber.

### 4.6. Western Blot

Forty-eight hours after transfection, cells were lysed in Radio-Immunoprecipitation Assay (RIPA) buffer (Beyotime, Shanghai, China). Equivalent amounts of lysates were loaded and separated by SDS-PAGE, and transferred onto polyvinylidene fluoride (PVDF) membranes. The membranes were blocked for 0.5 h at room temperature and incubated with specific primary antibodies overnight at 4 °C. Membranes were further incubated with corresponding Horseradish Peroxidase (HRP)-conjugated secondary antibodies for 1 h at room temperature, and blots were visualized using an ECL Kit (Millipore, Hayward, CA, USA).

### 4.7. Luciferase Reporter Assays

HEK-293 cells were cultured in 24-well plate and co-transfected with 100 nM of miR-143 or control mimics and WT or Mut using Lipofectamine 2000. Fourty-eight hours after transfection, cells were harvested, and luciferase activity was assayed using a Dual-Luciferase Reporter Assay kit (Promega, Wisconsin, WI, USA).

### 4.8. Statistical Analysis

All data were evaluated using SPSS 16. One-way ANOVA test or student’s *t* test was performed to analyze the significant differences. *p* < 0.05 was considered statistically significant.

## 5. Conclusions

Our data suggest that the frequently decreased miR-143 might lead to the increased Limk1 and in turn contribute to the development of NSCLC.
